# Insulin impedes osteogenesis of BMSCs by inhibiting autophagy and promoting premature senescence via the TGF-β1 pathway

**DOI:** 10.18632/aging.102723

**Published:** 2020-02-03

**Authors:** Ping Zhang, Hengguo Zhang, Jialin Lin, Tao Xiao, Rongyao Xu, Yu Fu, Yuchao Zhang, Yifei Du, Jie Cheng, Hongbing Jiang

**Affiliations:** 1 Jiangsu Key Laboratory of Oral Diseases, Nanjing Medical University, Nanjing 210029, Jiangsu Province, China; 2 Department of Oral and Maxillofacial Surgery, Affiliated Hospital of Stomatology, Nanjing Medical University, Nanjing 210029, Jiangsu Province, China

**Keywords:** insulin, TGF-β1, autophagy, osteogenesis, senescence

## Abstract

The dysfunction of bone marrow stromal cells (BMSCs) may be a core factor in Type 2 diabetes mellitus (T2DM) associated osteoporosis. However, the underlying mechanism is not well understood. Here, we delineated the critical role of insulin impeding osteogenesis of BMSCs in T2DM. Compared with BMSCs from healthy people (H-BMSCs), BMSCs from T2DM patient (DM-BMSCs) showed decreased osteogenic differentiation and autophagy level, and increased senescent phenotype. H-BMSCs incubated in hyperglycemic and hyperinsulinemic conditions similarly showed these phenotypes of DM-BMSCs. Notably, enhanced TGF-β1 expression was detected not only in DM-BMSCs and high-glucose and insulin-treated H-BMSCs, but also in bone callus of streptozocin-induced diabetic rats. Moreover, inhibiting TGF-β1 signaling not only enhanced osteogenic differentiation and autophagy level of DM-BMSCs, but also delayed senescence of DM-BMSCs, as well as promoted mandible defect healing of diabetic rats. Finally, we further verified that it was TGF-β receptor II (TβRII), not TβRI, markedly increased in both DM-BMSCs and insulin-treated H-BMSCs. Our data revealed that insulin impeded osteogenesis of BMSCs by inhibiting autophagy and promoting premature senescence, which it should be responsible for T2DM-induced bone loss, at least in part. These findings suggest that inhibiting TGF-β1 pathway may be a potential therapeutic target for T2DM associated bone disorders.

## INTRODUCTION

Diabetes mellitus (DM), as a chronic metabolic disease, is associated with several clinical complications, and affects the heart, blood vessels, eyes, kidneys, and nerves. It has also been increasingly recognized that DM adversely affects bone health due to the formation of detrimental bone microenvironment, such as elevated advanced glycation end product (AGE), reactive oxygen species (ROS) and inflammatory states [1–4]. Functional impairment of bone marrow stromal cells (BMSCs) in the diabetic microenvironment may be a crucial factor leading to DM associated bone loss [5–7]. These findings suggest that the dysfunction of BMSCs in the diabetic microenvironment may be attributed to aberrant bone metabolism. However, how insulin impedes the function of BMSCs, and the underlying mechanism is not well understood.

As the most common type of diabetes, type 2 diabetes mellitus (T2DM) begins with insulin resistance induced by hyperglycemia, and accordingly body cells fail to respond and uptake of insulin in the body [8, 9]. Hyperglycemia resulting from a failure of compensation for insulin resistance [10] is involved in the pathogenesis of diabetic bone disease on account of sustaining inflammation, which can inhibit osteoblast differentiation and function through inhibiting expression of the osteoblast marker genes [11–13]. There is a close relation between DM associated bone loss and dysfunction of BMSCs, but the detailed molecular and cellular mechanisms underlying the insulin impeding osteogenesis of BMSCs in T2DM remain largely unexplored.

Autophagy is an essential cellular metabolic pathway that sustains cytoplasmic homeostasis by eliminating damaged macromolecules and organelles under starvation or oxidative stress stimulation, which maintains cell survival and growth [14, 15]. Autophagy helps to recycle cell components and favor osteogenesis of BMSCs by scavenging reactive oxygen species (ROS) under harsh conditions, which maintain bone remodeling [14, 16, 17]. Autophagy defects have also been reported to have a strong relationship with DM associated inflammatory state and bone metabolic disorder [18, 19].

During the progression of T2DM, patients often show compensatory insulin overexpression for many years before declines. Previous studies have demonstrated that hyperinsulinemia reduces osteoblast activity via upregulation of transforming growth factor-β (TGF-β) and is associated with decreased bone metabolism-related gene expression, which subsequently impairs trabecular micro-architecture [20, 21]. TGF-β1 is released from the bone matrix and activated during osteoclast-mediated bone resorption [22]. Aberrant activation TGF-β1 was found in the subchondral bone in humans with osteoarthritis. Blockade of TGF-β signaling attenuated articular cartilage damage, delaying osteoarthritis onset [23]. Overexpression of TGF-β1 due to gene mutations is associated with skeletal diseases, such as Camurati-Engelmann disease (CED), which is an inherited skeleton remodeling disorder [24]. Notably, TGF-β1 is reportedly increased in the serum of DM patients [25, 26]. These findings raise an intriguing possibility that insulin-TGF-β1 may act as a regulatory pathway in dysfunction of BMSCs, which may be responsible for DM associated bone disorders.

In this study, we aimed to investigate the effect of hyperglycemia and hyperinsulinemia on the function of BMSCs and associated regulatory mechanisms. We compared osteogenic capacity and autophagy level, and senescent phenotype between BMSCs from T2DM patient (DM-BMSCs) and BMSCs from healthy people (H-BMSCs). Our results demonstrated that insulin-TGF-β1 pathway impeded osteogenesis of BMSCs by inhibiting autophagy and promoting premature senescence, indicating a previously unknown mechanistic linkage of insulin-TGF-β receptor II (TβRII) pathway in T2DM associated bone disorders.

## RESULTS

### DM-BMSCs show a decreased osteogenic differentiation and autophagy level, and an increased senescent phenotype

**Figure 1:**
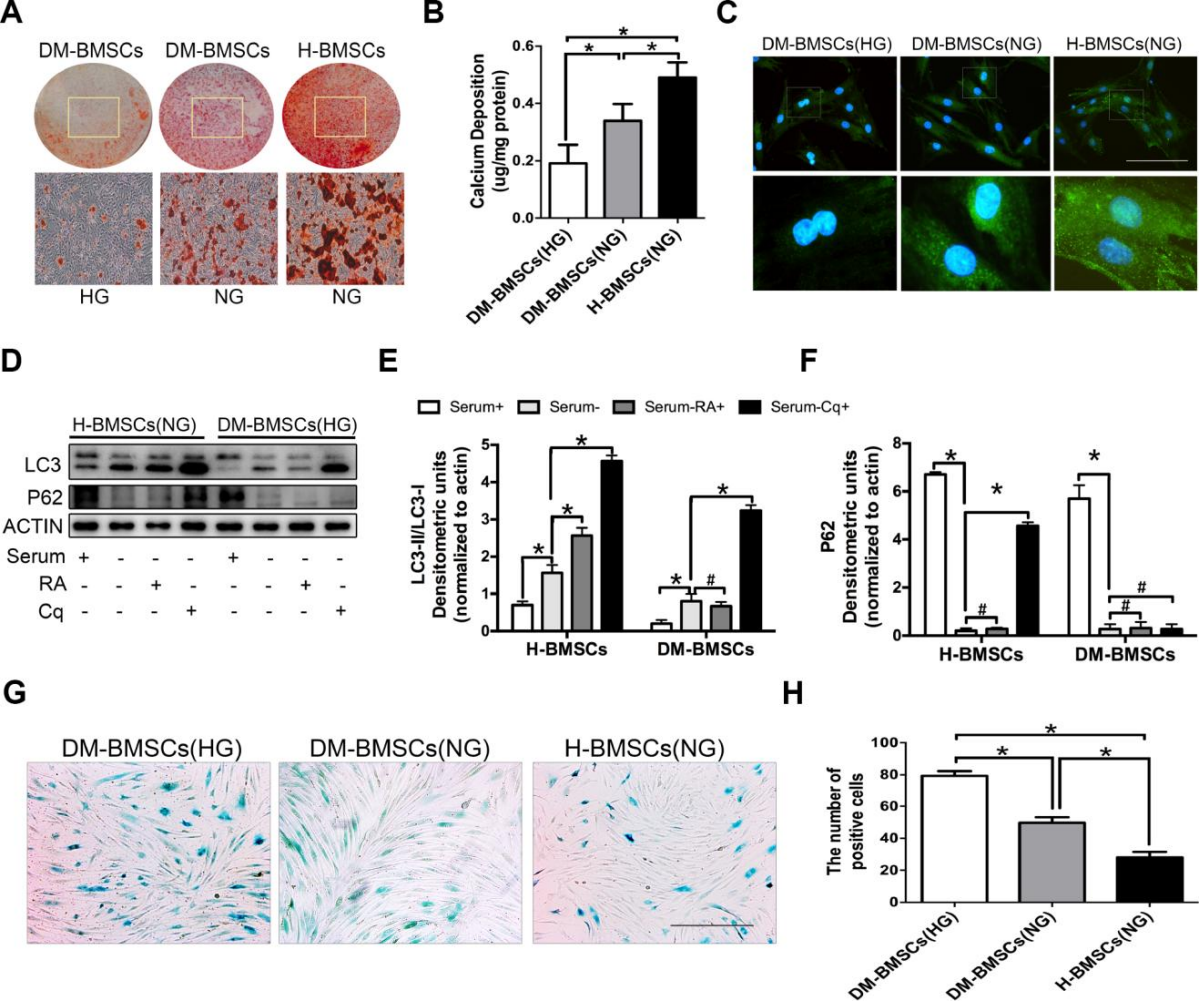
**DM-BMSCs show a decreased osteogenic differentiation and autophagy level, and an increased senescent phenotype.** DM-BMSCs and H-BMSCS were incubated in the corresponding condition and treated by osteogenic inducement for 14 days and stained with Alizarin Red S (**A**). Quantitative analysis of the amount of calcium-bound stain was determined by comparison with calcium standards (**B**). Fluorescence detection of autophagosomes in DM-BMSCs and H-BMSCs transfected with the GFP-LC3 plasmid and cultured in serum deprivation conditions for 6 h (**C**). The expression of LC3 and P62 were detected by western blot after serum deprivation for 6 h (**D**). Protein bands were quantified and analyzed by densitometric analysis. Rapamycin (RA) as positive control and chloroquine (Cq) as negative control (**E**, **F**). Cell senescence was detected by SA-β-Gal staining after incubation in the corresponding condition (**G**). The number of positive cells was calculated (**H**). NG, normoglycemic condition, HG, hyperglycemic condition. Data are presented as the mean ± standard deviation, n=3. *p<0.05, ^#^p>0.05. Scale bar = 100 μm.

**Figure 2:**
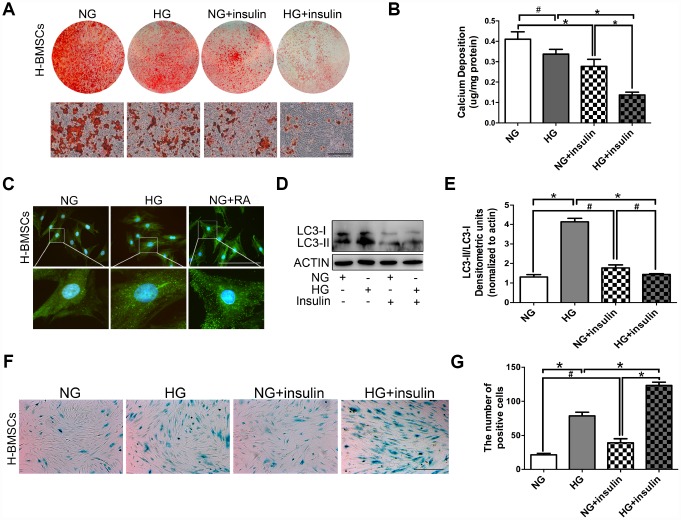
**Insulin impedes osteogenic differentiation of H-BMSCs by inhibiting autophagy and inducing senescence.** H-BMSCs were incubated under normoglycemic or hyperglycemic conditions with or without of insulin. The amount of calcium accumulation was measured by Alizarin Red S staining after osteogenic inducement for 14 days (**A**). Quantitative analysis of calcium-bound staining was determined by comparison with calcium standards (**B**). H-BMSCs were cultured in normal or high-glucose medium. Rapamycin (RA) as positive control. Fluorescence detection of autophagosomes in H-BMSCs were transfected with the GFP-LC3 plasmid and cultured in serum deprivation conditions for 6 h (**C**). H-BMSCs cultured in normal or high-glucose medium with or without insulin. Western blot analysis showed the conversion of LC3-I into LC3-II after serum deprivation for 6 h (**D**). Protein bands were quantified and analyzed by densitometric analysis (**E**). H-BMSCs cultured in normal or high glucose medium with or without insulin for 3 days. H-BMSC senescence was measured by SA-β-Gal staining (**F**). The number of positive cells was calculated (**G**). NG, normoglycemic condition, HG, hyperglycemic condition. Data are presented as the mean ± standard deviation, n=3. *p<0.05, ^#^p>0.05. Scale bar = 100 μm.

To simulate the pathophysiological status, DM-BMSCs and H-BMSCs were respectively incubated under hyperglycemic or normoglycemic conditions. After 14 days of osteogenic induction, Alizarin Red S stain showed a significant difference between hyperglycemic conditions and normoglycemic conditions in DM-BMSCs, but not in H-BMSCs (Figure 1A, 1B, Figure 2A, 2B). Compared with H-BMSCs, the less calcifying nodules staining was observed in DM-BMSCs regardless of culture conditions. Interestingly, the more calcifying nodules staining was observed in DM-BMSCs under normoglycemic conditions compared with the hyperglycemic conditions (Figure 1A, 1B). These results indicated a decreased osteogenic differentiation of DM-BMSCs.

We compared autophagy levels between DM-BMSCs and H-BMSCs by imaging GFP-LC3b-labeled BMSCs. The results revealed that DM-BMSCs weakly expressed LC3 after 6 hours of serum deprivation compared with H-BMSCs (Figure 1C). To explore which stage of autophagy was suppressed, we detected the expression of LC3 and P62 in DM-BMSCs and H-BMSCs after using rapamycin (RA, autophagy activator at the early stagy) or chloroquine (Cq, autophagy inhibitor at the late stagy). The results indicated the LC3-II/LC3-I ratio increased, and the expression of P62 decreased in both DM-BMSCs and H-BMSCs after serum deprivation, but the expression of LC3-II in DM-BMSCs was less than that in H-BMSCs. After addition of RA, H-BMSCs showed an increased LC3-II/LC3-I ratio, but DM-BMSCs did not show a similar change. Moreover, after addition of Cq, P62 expression in H-BMSCs increased significantly, but it did not in DM-BMSCs (Figure 1D–1F). These results suggested that the autophagy process of DM-BMSCs was suppressed at the early stage, which then led to a decreased autophagy levels. We also compared the senescence phenotype between DM-BMSCs and H-BMSCs by senescence-associated β-galactosidase (SA-β-Gal) staining and found more SA-β-Gal-positive cells in DM-BMSCs than in H-BMSCs (Figure 1G, 1H). Together, these data indicated that DM-BMSCs showed a decreased osteogenic differentiation and autophagy levels, and an increased senescent phenotype.

### Insulin impedes osteogenic differentiation of H-BMSCs by inhibiting autophagy and promoting senescence

H-BMSCs were incubated under normoglycemic or hyperglycemic conditions with or without 200 μIU/ml of insulin. After 14 days of osteogenic induction, Alizarin Red S staining did not show a significant difference between normoglycemic conditions and hyperglycemic conditions, but less calcifying nodules staining was observed in normoglycemic conditions with insulin, and the least calcifying nodules was observed in hyperglycemic conditions with insulin (Figure 2A, 2B). These results suggested that insulin inhibited osteogenic differentiation of BMSCs, and this trend is especially apparent under hyperglycemic conditions.

To detect the potential role of autophagy in insulin impeding osteogenic differentiation of BMSCs, GFP-LC3b-labeled H-BMSCs were cultured in normal or high-glucose medium. After 6 hours of serum deprivation, H-BMSCs in hyperglycemic conditions expressed more LC3 than that in normoglycemic conditions (Figure 2C). The expression of LC3-II and LC3-II/LC3-I ratio was higher in hyperglycemic conditions but significantly decreased in the group of insulin addition (Figure 2D, 2E). In addition, SA-β-Gal staining showed that more senescent cells were observed in hyperglycemic conditions than in normoglycemic conditions, and the most obvious senescent phenotype was found in high-glucose and insulin conditions (Figure 2F, 2G). These data indicated that insulin impeded osteogenic differentiation of H-BMSCs by inhibiting autophagy and promoting senescence.

### TGF-β1 participates in insulin inhibiting autophagy and osteogenic differentiation of H-BMSCs

**Figure 3:**
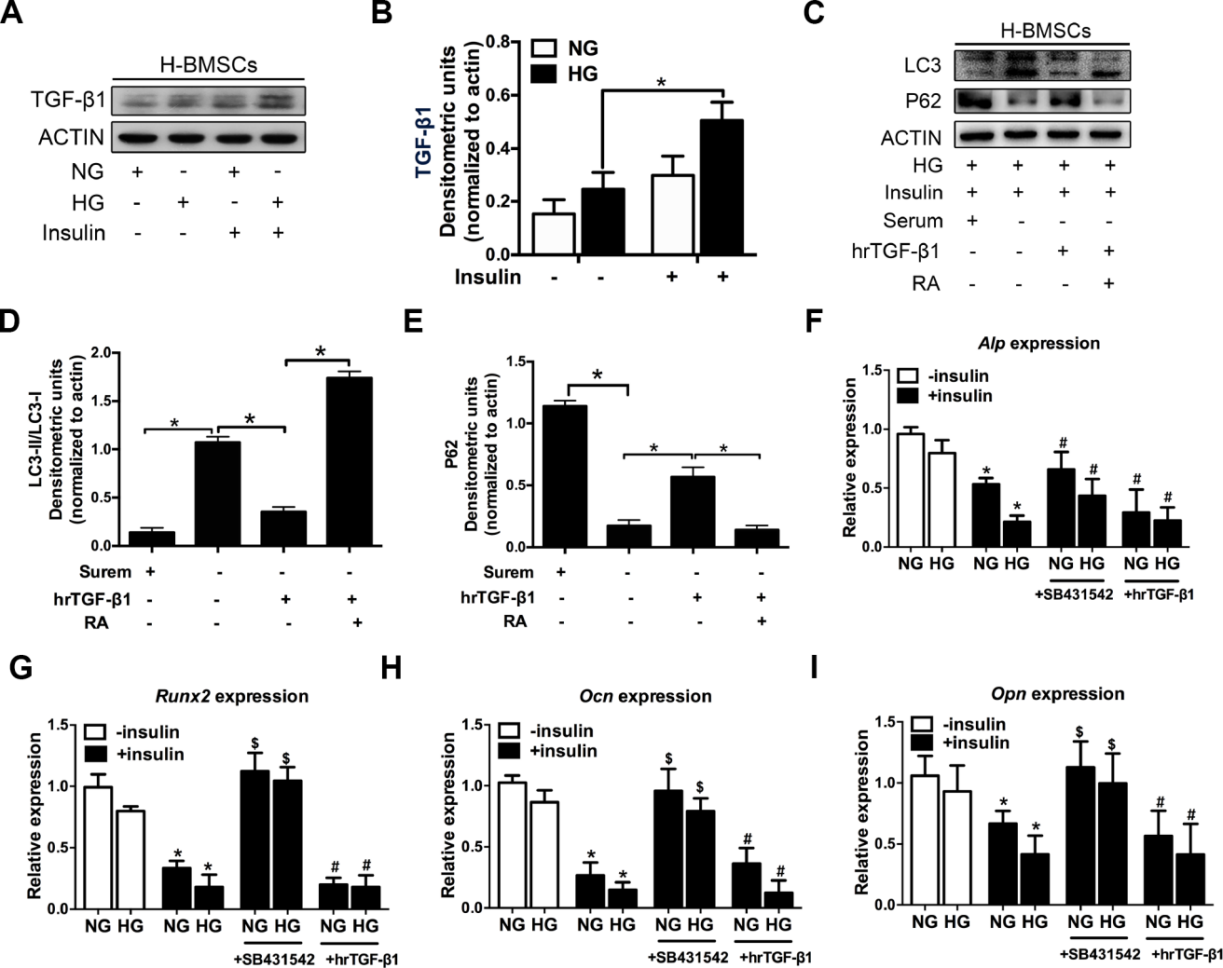
**TGF-β1 participates in insulin inhibiting autophagy and osteogenic differentiation of H-BMSCs.** H-BMSCs were cultured in normal or high-glucose medium with or without insulin. The expression of TGF-β1 in H-BMSCs was detected by western blot (**A**). Protein bands were quantified and analyzed (**B**). H-BMSCs were incubated in hyperglycemic and insulin conditions with or without hrTGF-β1. Rapamycin (RA) as positive control. The protein level expression of LC3 and P62 were detected by western blot (**C**). Protein bands were quantified and analyzed by densitometric analysis (**D**, **E**). H-BMSCs were cultured in osteogenic medium for 7 days with or without insulin under normoglycemic or hyperglycemic conditions stimulated with SB431542 or hrTGF-β1. mRNA level expression of *Alp* (**F**), *Runx2* (**G**), *Ocn* (**H**), and *Opn* (**I**) was detected by real-time PCR. NG, normoglycemic condition, HG, hyperglycemic condition. Data are presented as the mean ± standard deviation, n=3. *p<0.05, ^$^p<0.05, ^#^p>0.05, * in F-I as compared to insulin-untreated cells, ^$^ and ^#^ in F-I as compared to corresponding insulin-treated cell.

Previous research indicated that hyperinsulinemia reduces osteoblast activity via upregulation of TGF-β [20], and these results prompted us to investigate the potential role of TGF-β1 in insulin-mediated osteogenic differentiation of H-BMSCs. As expected, TGF-β1 expression increased in H-BMSCs under hyperglycemic and hyperinsulinemia conditions (Figure 3A, 3B). To investigate the role of TGF-β1 in insulin inhibiting autophagy, human recombinant active TGF-β1 (hrTGF- β1) was added, and the results showed that the expression of LC3-II and LC3-II/LC3-I ratio significantly decreased, while the expression of P62 increased. In addition, RA reversed these effects of hrTGF-β1 (Figure 3C–3E). These data suggested that TGF-β1 accelerated autophagy suppression of H-BMSC under high-glucose and insulin conditions.

Next, we investigated whether TGF-β1 participated in insulin-inhibiting osteogenic differentiation of H-BMSC. After 7 days of osteogenic induction with addition of hrTGF-β1 or TGF-β type I receptor/ALK5 inhibitor SB431542, the results showed that the expression of *Alp*, *Runx2, Ocn, and Opn* decreased in insulin groups, but these genes except *Alp* increased in addition of SB431542 (Figure 3F–3I). These data indicated that insulin inhibited osteogenic differentiation of H-BMSC in a TGF-β1 pathway dependent manner.

### TGF-β1 promotes senescence, inhibits autophagy and osteogenic differentiation of DM-BMSCs

**Figure 4:**
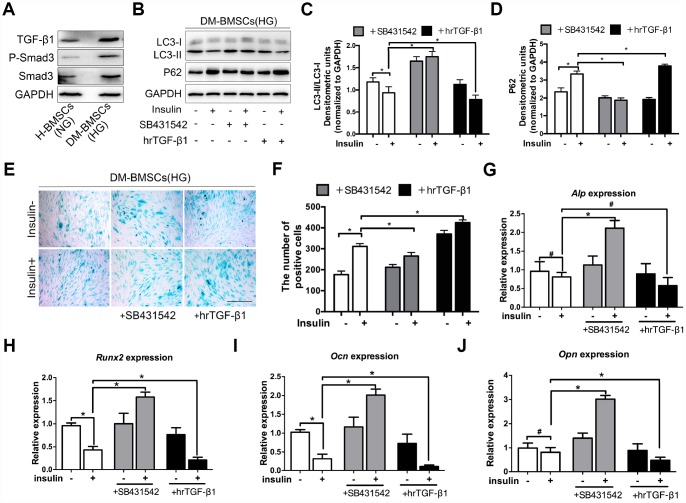
**TGF-β1 promotes senescence, inhibits autophagy and osteogenic differentiation of DM-BMSCs.** DM-BMSCs were incubated under hyperglycemic conditions, and H-BMSCS were incubated under normoglycemic conditions. The expression of the TGF-β1 signaling pathway was measured by western blot (**A**). DM-BMSCs were incubated under hyperglycemic conditions with or without insulin addition of the SB435142 or hrTGF-β1 for 3 days. The expression of LC3 and P62 were detected by western blot after serum deprivation for 6 h (**B**). Protein bands were quantified and analyzed by densitometric analysis (**C**, **D**). Cellular senescence was detected by SA-β-Gal staining after incubation in the corresponding condition for 3 days (**E**). The number of positive cells was calculated (**F**). DM-BMSCs were cultured in osteogenic medium for 7 days with or without insulin under hyperglycemic conditions stimulated with SB431542 or hrTGF-β1. mRNA level expression of *Alp* (**G**), *Runx2* (**H**), *Ocn* (**I**), and *Opn* (**J**) was detected by real-time PCR. NG, normoglycemic condition, HG, hyperglycemic condition. Data are presented as the mean ± standard deviation, n=3. *p<0.05, ^#^p>0.05. Scale bar = 100 μm.

Insulin impedes osteoblast differentiation, inhibits autophagy and promotes premature senescence of H-BMSCs in a TGF-β1 dependent manner. To further explore whether these effects of insulin also present in the DM-BMSCs, we first examined the expression of the TGF-β1 signaling pathway in DM-BMSCs, and found the expression of TGF-β1 and P-smad3 increased in DM-BMSCs (Figure 4A). Then, we investigated autophagy, senescence, and osteogenic differentiation of DM-BMSCs by inhibiting or activating the TGF-β1 signal pathway. The results showed that the LC3-II/LC3-I ratio was decreased and the P62 was increased in insulin-treated DM-BMSCs compared with untreated cells. However, when blocking the TGF-β1 signal with SB431542, the LC3-II/LC3-I ratio increased and P62 decreased. Conversely, the LC3-II/LC3-I ratio decreased and P62 increased when activating the TGF-β1 signal with hrTGF-β1 (Figure 4B–4D). These results suggested that insulin inhibited autophagy, promoted senescence of DM-BMSCs and these effects could be abrogated by inhibiting TGF-β1 signaling.

To investigate the effect of TGF-β1 signaling on senescence and osteogenic differentiation of DM-BMSCs, SB431542 or hrTGF-β1 was respectively employed to inhibit or activate TGF-β1 signaling. The results showed more SA-β-Gal-positive cells in the presence of insulin compared with the absence of insulin. The positive cells decreased in SB431542 group, whereas increased in hrTGF-β1 group (Figure 4E, 4F). Next, we investigated whether TGF-β1 participated in insulin-inhibiting osteogenic differentiation of DM-BMSC, and found that the expression of *Run2*, *Ocn*, and *Opn* increased when TGF-β1 signaling was blocked, whereas these genes decreased when TGF-β1 signaling was activated (Figure 4G–4J). These data indicated that senescence and osteogenic differentiation of insulin-inhibiting DM-BMSCs was promoted or attenuated with the change of TGF-β1 signaling.

### Suppression of TGF-β1 signaling promotes repair of mandible defects in diabetic rat

**Figure 5:**
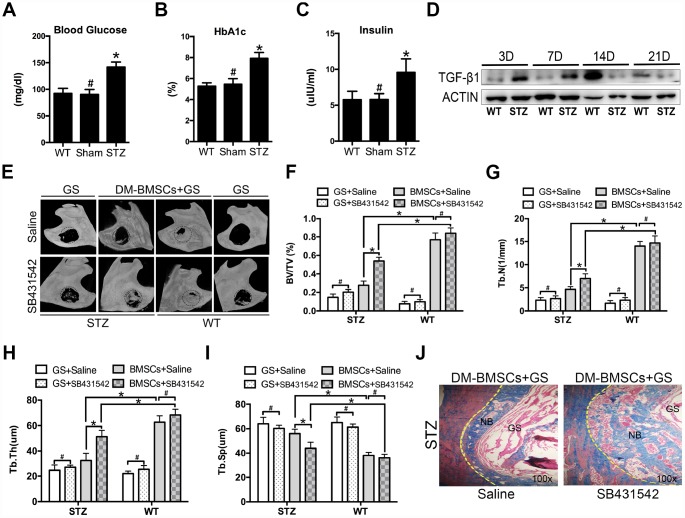
**Suppression of TGF-β1 signaling promotes repair of mandible defects in diabetic rat**. Blood was collected from the tail veins after overnight fasting, and blood glucose levels were measured using a glucometer (**A**). Blood samples were taken from anesthetized rats through retrobulbar venous plexuses. Serum insulin and HBA1c levels were measured in a clinical laboratory (**B**, **C**). Full thickness bone defect (1×3mm) was made in the mandibular margins, and calluses were harvested at postoperative 3, 7, 14, and 21 days. The expression of TGF-β1 was measured by western blot (**D**). Mandibular critical-sized bone defects (diameter 5mm) were made in an STZ-induced diabetic rat and WT rat model, and human DM-BMSCs with gelatin sponge (GS) or GS alone were implanted into defects. SB431542 or saline were injected into the local defect once every 2 days for 1 week. After 8 weeks, micro-CT images were taken (**E**). Trabecular bone volume fraction (BV/TV), trabecular number (Tb.N.), trabecular thickness (Tb.Th.), trabecular separation (Tb.Sp.) were analyzed (**F**–**I**). New bone formation in STZ group implantation of DM-BMSCs with GS were evaluated by Masson trichrome staining (**J**). Black dotted lines in E indicate the size and location of defect. Yellow dotted lines in J indicate the bonder of defect. NB, new bone, GS, gelation sponge. Data are presented as the mean ± standard deviation, n=3. *p<0.05, ^#^p>0.05.

To mimic the whole features of early stage of type 2 diabetes, HFD and STZ were used to induce peripheral insulin resistance. The STZ group showed significantly higher plasma glucose, HBA1c and insulin levels compared to sham and WT group (Figure 5A–5C). Full thickness bone defects (1×3mm) were made in the inferior border of the mandible, and the calluses were harvested at postoperative 3, 7, 14, and 21 days. The results showed the expression of TGF-β1 increased in STZ group (Figure 5D). To investigate whether blocking the TGF-β1 signaling could promote bone regeneration, mandibular critical-sized bone defects were made in diabetic rat and wide type rat. DM-BMSCs with gelatin sponge (GS) or GS alone were implanted into defects. SB431542 or saline were injected into the local defect once every 2 days for 1 week. After 8 weeks, GS group did not show any new bone formation on the saline side, while a small amount of new bone was showed on the SB431542 side both in STZ and WT rat. DM-BMSC and GS group showed more plate-like bone structures on the SB431542 side than the saline side in STZ rat, and no significant difference was displayed on the two sides of WT rat (Figure 5E). The quantitative analysis indicated that the ratio of new bone volume to total volume (BV/TV), the number of trabecular bone (Tb.N.) and trabecular thickness (Tb.Th.) were higher on the SB431542 side than the saline side (Figure 5F–5H). However, there was less trabecular separation (Tb.Sp.) on the SB431542 side than the saline side (Figure 5I). Histological results also showed more new bone on the SB431542 side, displaying a large quantity of organized and mineralized bone tissue with lamellar bone morphology (Figure 5J). These results indicated that suppression of TGF-β1 signaling could promote bone regeneration in diabetic rat.

### Insulin promotes TGF-β1 receptor II expression

**Figure 6:**
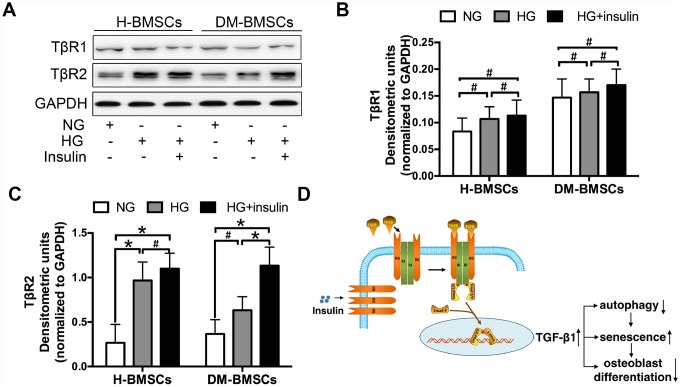
**Insulin promotes TGF-β1 receptors II expression.** H-BMSCs and DM-BMSCs were cultured in normoglycemic or hyperglycemic medium with or without insulin. The expression of TGF-β1 receptors was detected by western blot (**A**). Protein bands were quantified and analyzed by densitometric analysis (**B**, **C**). A schematic working model for insulin function in the regulation of autophagy, senescence and osteoblast differentiation via mobilization of TGF-β1 receptor II in cell surface (**D**). NG, normoglycemic condition, HG, hyperglycemic condition. Data are presented as the mean ± standard deviation, n=3. *p<0.05, ^#^p>0.05.

To explore the underlying mechanisms of TGF-β1signaling, we detected the expression of TGF-β1 receptor I and II (TβRI, TβRII) in H-BMSCs and DM-BMSCs. The results demonstrated that TβRII expression increased in H-BMSCs or DM-BMSCs under hyperglycemic and hyperinsulinemia conditions (Figure 6A, 6C), but TβRI expression did not change in these conditions (Figure 6A, 6B). These results suggested that insulin increased the expression of TGF-β1 receptor II, which was most likely responsible for the activation of the TGF-β1 signaling pathway. Thus, we conclude that insulin induces a modest increase of TβRII expression and then activates the TGF-β1 signaling pathway driving the downstream insulin-responsive genes activation, which impede osteogenesis of BMSCs by inhibiting autophagy and promoting premature senescence (Figure 6D).

## DISCUSSION

The progression of T2DM is considered to be associated with alterations in blood glucose and insulin levels. It is well known that BMSCs play crucial key in maintaining bone mass [27]. Therefore, to understand T2DM associated bone disorders and the underlying mechanisms, we investigated the effect of glucose and insulin on the physiological functions of BMSCs. Our data revealed that a decreased osteogenic differentiation and autophagy activity, and an increased premature senescence of DM-BMSCs, and similar results were also found in H-BMSCs under hyperglycemic or hyperinsulinemia conditions.

High glucose-inducing stress at the cellular level and glycating proteins lead to the formation of advanced glycation end products and increase production of reactive oxygen species, and these factors are considered to be the primary cause of cellular senescence [2, 28]. In addition, autophagy is a protective mechanism for maintaining cellular physiology and promoting survival in a harsh environment. Our results indicated that autophagy activity was beneficial for H-BMSC survival in high-glucose conditions, but autophagy activity was suppressed when insulin was added simultaneously, resulting in a premature senescence phenotype of H-BMSC. The existing evidence indicates that insulin inhibits autophagy in many ways, such as activating mammalian target of rapamycin (mTOR) in synergy with amino acids [29], Akt-mediated phosphorylation and inhibition of the transcription factor FoxO3 [30], and inhibiting expression of autophagy-related genes, such as VPS34 and Atg12 in a FoxO1-dependent manner [31]. Therefore, insulin-induced autophagy suppression and premature senescence may be responsible for the poor osteogenic differentiation of H-BMSCs under hyperglycemic and hyperinsulinemia conditions.

Previous studies have demonstrated that hyperinsulinemia reduces osteoblast activity by TGF-β pathway, and there exists a positive correlation between hrTGF-β1 stimulation and dysfunction of osteoblast [20, 32]. Consistent with these results, TGF-β1 was significantly upregulated in H-BMSCs cultured in high-glucose and insulin conditions. And then, we asked whether insulin affected H-BMSCs in a TGF-β1-dependent manner, the results showed that insulin-inhibiting autophagy of H-BMSC was further suppressed in the presence of hrTGF-β1. Notably, insulin-inhibiting osteogenic differentiation of H-BMSCs could be restored when TGF-β signaling was blocked. Similar results were also verified in the DM-BMSCs. Importantly, suppression of TGF-β1 signaling could promote bone regeneration in diabetic rats.

TGF-β drives its function through cell surface receptor complexes of TβRI and TβRII. Following ligand binding, the TβRII receptors phosphorylate and activate the TβRI receptors, which C-terminally phosphorylate and thereby activate Smad2 and Smad3, then forming a complex with Smad4, translocating into the nucleus and regulating the transcription of TGF-β-responsive genes [33, 34]. In diabetic patients and rodent models of diabetes, continuous exposure of cells to high glucose has been found to increase TGF-β1 levels in the glomerular and tubular compartments of the kidney, and Smad3 activation was observed in these cells [35–37]. High glucose was also shown to induce TGF-β1 expression and/or the TβRII receptor [36, 38]. Furthermore, high glucose induced a rapid increase of TβRI and TβRII at the cell surface without changing their total expression and thus confers increased TGF-β responsiveness [39]. These data suggest a functional linkage of glucose with increased TGF-β1 signaling.

Insulin is released by pancreatic β cells and induces glucose uptake by decreasing the glucose levels in blood. Because of its central role in glucose homeostasis, insulin administration is the standard therapy for hyperglycemia in diabetic patients. Insulin can also induce a rapid increase of the TGF-β receptors TβRI and TβRII at the cell surface [40]. Insulin-induced TGF-β receptor abundance enhanced the cell responsiveness to autocrine TGF-β, with increased TGF-β/Smad signaling consequently participating in the cellular response to insulin. Thus, increased TGF-β responsiveness and signaling activation are integral to the insulin-responsive gene expression and allows for cooperation between TGF-β1 and insulin signaling. These findings highlight a role of insulin in controlling the TGF-β1-receptor availability and TGF-β1 responsiveness, and it may explain the effects of insulin on BMSCs in a TGF-β1-dependent manner.

TGF-βs are synthesized as large precursor molecules, composed of mature TGF-β and latency-associated protein (LAP). LAP remains noncovalently bound to mature TGF-β as it is secreted to render it inactivated [41, 42]. TGF-β1 is one of the most abundant cytokines in the bone matrix [43, 44]. During tissue injury or bone remodeling, TGF-β1 in the bone matrix can be activated by cleavage of LAP via osteoclasts [45]. A gradient of active TGF-β1 then signals to transiently recruit perivascular BMSCs to the recently resorbed bone surface for osteoblast differentiation and new bone formation. The gradient of TGF-β1 generated at the resorption sites likely prohibits further recruitment of osteoclast precursors, and protects it from further resorption during the reversal phase of bone remodeling. High levels of TGF-β1 in the bone marrow and abnormalities in bone remodeling are associated with multiple skeletal disorders, such as CED, characterized by a fusiform thickening of the diaphysis of the long bones and skull, and caused by mutations in *TGFB1* that result in premature activation of TGF-β1 [46–48].

In diabetes, more TGF-β1 and many other bone matrix factors were released to the marrow and blood during the bone healing due to increased osteoclastogenesis [3, 49]. Our results indicated that TGF-β1 activation at the early stage of bone healing in the STZ-induced diabetic rat model. An increased TGF-β ligand in the microenvironment may be an important factor for upregulation of TGF-β signaling, and TβRII upregulation on the cell surface may be another, which enhance the cell responsiveness to TGF-β ligand. Thus, we conclude that the insulin-induced TβRII expression on the cell surface promotes BMSC responsiveness to the TGF-β ligand in the microenvironment, which are integral to the insulin-responsive gene expression.

In order for bone homeostasis to be maintained through adulthood, bone remodeling must be consistently sustained in space and time. TGF-β1 is one of the key cytokines responsible for coupling bone resorption with formation. In diabetes, insulin-induced TGF-β1 upregulation by mobilization of the TGF-β type II receptor in DM-BMSCs are responsible for diabetes-related bone healing disorder. Therapies that can attenuate TGF-β1 signaling may serve as potential therapies for these bone disorders.

## MATERIALS AND METHODS

### Ethical statement

All research on human subjects follows the Helsinki Declaration. All experiments were performed with the approval of the Ethics Committee of the Nanjing Medical University (Approval number: 2015-52). All participants signed an informed consent before entering the study to use their tissues for research purposes. The demographic data and the specified parameters of donors were showed in Supplementary Table 1. Bone marrow stem cells were derived from the jaw bones of healthy people (H-BMSCs) and T2DM (DM-BMSCs) undergoing dental implant surgery or tooth extraction. Isolation and culture of BMSCs from the mandible have been previously described [50]. BMSCs were Spindle-shaped adherent cells, and could develop the colony-forming units and undergo the osteoblast differentiation in osteogenic medium [50]. Screening criteria for T2DM conform to ABC or D. (A) Fasting blood glucose (FBG) > 6.0 mmol/L, (B) glycated glycemic protein (HBA1c) > 6.5%, (C) fasting insulin > 10 mIU/L, (D) fasting blood glucose (mmol/L) × fasting insulin (mIU/L) > 60.

### Cell culture medium

Normal glucose (NG): Dulbecco’s modified Eagle’s medium (DMEM) (1.0 g/L D-glucose, Gibco, Grand Island, NY, USA), 10% fetal bovine serum (FBS, ScienCell, Carlsbad, CA, USA), 100 U/ml of penicillin (Gibco Life Technologies, Grand Island, NY, USA), and 100 μg/ml of streptomycin (Gibco Life Technologies, Grand Island, NY, USA)

High glucose (HG): DMEM (4.5g/L D-glucose, Gibco, Grand Island, NY, USA), 10% FBS, 100 U/ml of penicillin, 100 μg/ml of streptomycin NG+insulin: DMEM (1.0g/L D-glucose), 10% FBS, 100 U/ml of penicillin, 100 μg/ml of streptomycin, 200 μIU/ml of insulin (Jiangsu Wanbang Biochemical Pharmaceutical Co., Ltd. Nanjing, China) HG+Insulin: DMEM (4.5g/L D-glucose), 10% FBS, 100 U/ml of penicillin, 100 μg/ml streptomycin, 200 μIU/ml of insulin HG+H_2_O_2_: DMEM (1.0g/L D-glucose), 10% FBS, 100 U/ml penicillin, 100 μg/ml streptomycin, 100 μM H_2_O_2_ HG+RA: DMEM (4.5g/L D-glucose), 10% FBS, 100 U/ml of penicillin, 100 μg/ml of streptomycin, and 100 nM of rapamycin (RA) HG+Insulin+TGF-β1 inhibitor: DMEM (1.0g/L D-glucose), 10% FBS, 100 U/ml of penicillin, 100 μg/ml of streptomycin, 200 μIU/ml of insulin, 5 μM of TGF-β1 inhibitor (Alk5 inhibitor, Alk5i, SB431542, Sigma, St. Louis, USA) HG+Insulin+human recombinant cytokine TGF-β1: DMEM (1.0g/L D-glucose), 10% FBS, 100 U/ml penicillin, 100 μg/ml streptomycin, 200 μIU/ml of insulin, 5 ng/ml of human recombinant cytokine TGF-β1 (hrTGF-β1) (Peprotech, London, UK)

### Real time polymerase chain reaction (PCR)

RNA was extracted from a single 25cm^2^ flasks of cells using Trizol reagent and reverse transcribed using the PrimeScript RT Reagent Kit (Takara Bio, Kusatsu, Japan). Real-time quantitative PCR analyses were performed in triplicate using the SYBR Green PCR Master Mix (Takara Bio) and detected using an Applied Biosystems 7300. RNA extraction and real-time PCR analysis were performed as previously described [50]. The primer sequences used in this study are shown in Supplementary Table 2.

### Western blot and immunofluorescence staining

Western blots and immunofluorescence staining were performed according to our previous procedures [50]. Briefly, a single 25cm^2^ flasks of cell lysates were separated on polyacrylamide–sodium dodecyl sulfate gel and electroblotted onto nitrocellulose membranes (Bio-Rad, Hercules, CA, USA). After blocking with 5% nonfat dry milk, the membranes were incubated with various antibodies overnight. The primary antibodies used for Western blot and immunofluorescence staining were listed in Supplementary Table 3. Western blots were quantitatively analyzed using Quantity One image analysis software (Version 4.4, Bio-Rad) and then normalized using β-actin or GAPDH protein as an internal standard.

### Autophagy assay

Autophagy was induced by serum deprivation. Autophagy analysis was performed as previously described [51]. BMSCs were seeded into 6-well plates, at a density of 1×10^5^ cells/well. BMSCs stably expressing green fluorescent microtubule-associated protein 1 light chain 3 (GFP-LC3b) were generated by plasmid transfection with pEGFP-LC3b (Addegen, Cambridge, MA, USA) and Lipofectamine-2000 (Gibco, Grand Island, NY, USA) followed by G418 (Sigma) selection. The expression of the autophagy markers LC3 and P62 were examined by both Western blotting and immunofluorescence cytochemistry. Rapamycin (Selleck Chemicals, Houston, USA) or Chloroquine (Selleck Chemicals, Houston, USA) were used as autophagy activators or inhibitors.

### SA-β-gal staining

Cells were seeded into 12-well plates, at a density of 1×10^4^ cells/well, and were cultured for 3 days, and the cell aging phenotype was detected by a β-galactosidase Staining Kit (GenMed Scientifics Inc., Shanghai, China) according to the manufacturer’s recommendations. In the view of enlarging to 200 times, 3 visual fields were selected randomly to take photos for statistics.

### Osteogenic induction and Alizarin Red staining

Cells were cultured in 12-well plates at a density of 1×10^4^ cells/well for 3 days. Then, the growth media were changed with induction medium supplemented with 50 μg/ml ascorbic acid (Sigma) and 10 mM β- glycerophosphate (Sigma) in the presence of 10^−7^ M dexameth- asone (Sigma). At 14 days, cells were fixed with 70 % ethanol, stained with 2 % Alizarin Red S (pH 4.2; Sigma) and viewed under a light microscope (Carl Zeiss, Germany). Calcium-bound stain was extracted with 0.5 NaHCl/5 % sodium dodecyl sulfate, and the absorbance at 415 nm was compared with calcium standards as described previously [50].

### Animal experiments

Forty-eight male Sprague-Dawley rats, aged 10 weeks and weighing 180 to 250g were housed in a controlled environment (24±1°C, 12 h light:12 h dark cycle) and allowed food and water ad libitum. All procedures were carried out according to the guidelines of the Animal Care Committee of Nanjing Medical University. After 3-day acclimatization, 48 of the rats were randomly divided into 2 groups: the normal control group (WT group, n=18) and the high-fat diet group (HFD group, n=30). To induce an insulin-resistant (IR) model, the normal control (NC) group was fed by standard pellet animal diet, and the high-fat diet (HFD) group received HFD (mixing 20% sucrose, 3% yolk, 18% lard oil, and 59% standard pellet animal diet). After 4 weeks, rats were injected intraperitoneally (IP) with streptozotocin (STZ) (35 mg/kg, STZ group, n=24), freshly dissolved in 0.1 M citrate buffer or equal amount of saline (Sham group, n=6). After an additional week (the 5^th^ week from the starting of the experiment), the animals that showed stable random hyperglycemia (FBS ≥ 300 mg/dl) and index for insulin resistance (homeostatic model assessment of insulin resistance, HOMA-IR) more than 2.68 were involved in the study. The HFD was supplied until the end of the study. Fasting blood glucose levels were measured using a glucometer (Roche Diagnostics Products (Shanghai) Co., Ltd., Shanghai, China). Whole blood was collected from the carotid artery after catheter examination. Serum insulin and HBA1c levels were measured by a clinical laboratory (Affiliated Stomatological Hospital of Nanjing Medical University, Nanjing, China). Insulin resistance was estimated by the homeostasis model assessment index for insulin resistance (HOMA-IR) which was calculated as FBG (mmol/L) × FSI (μIU/ml)/22.5.

### Bone defect model construction

The rats were anesthetized by injection of pentobarbital (3.5 mg/100 g). An incision was made in the skin, and the mandible was exposed by dissection. Full thickness defects (1×3 mm) in both sides of the inferior border of the mandible were created in the STZ and WT groups. Calluses were harvested 3, 7, 14, and 21 days (n=3). Critical defects of 5 mm diameter in the ascending ramus of the mandible behind the root of the incisor were created in the STZ group using a low-speed dental engine with a bur that was continuously cooled by irrigation with a 0.9 % saline solution. Cells were trypsinized and harvested, washed twice with PBS solution, and re-suspended at 2.0 × 10^7^cells/ml in serum-free DMEM medium. Then, cells were seeded onto scaffolds by pipetting the cells suspension onto a gelatin sponge (GS) (Gelfoam; Upjohn, USA). Approximately 10^7^ cells were used per mandibular defect. The cells/scaffold construct was incubated for an additional 2 hours to allow cell attachment before implantation. The STZ and WT group were randomly divided into 2 groups that received one of the following implants: DM-BMSCs with gelatin sponge (GS) (n=6), or GS alone (n=6). Then, the incision was closed in layers with sutures. SB431542 was injected into the left side defect, and saline was injected into the right side defect once every 2 days for 1 week. After 8 weeks, micro-CT images and histological examination were taken.

### Micro-CT measurement and Masson staining

Micro-CT analysis and Masson staining were performed as previously described [51, 52]. The parameters of trabecular bone volume fraction (BV/TV), trabecular number (Tb.N.), trabecular thickness (Tb.Th.), and trabecular separation (Tb.Sp.) were used for comparison in this study.

### Digital image analysis

A total of 5 fields selected from area of interest (400X objective lens) were acquired per slide using a light microscope (Leica Microsystems, Mannheim, Germany). All photographs were randomly taken under the same conditions, including light source, color saturation, brightness, gain, and contrast. The photographs were then quantified using Image-Pro Plus 6.0 software. The integrated optical density (IOD) of all the positive staining in each field and area of interest (AOI) was measured. The IOD was used to evaluate the area and intensity of the positive staining. The mean density (IOD/AOI) represented the concentration of specific protein per unit area.

### Statistical analysis

All in vitro experiments were repeated independently at least 3 times. Results were presented as mean ± standard deviation (SD). The statistical significance between groups was calculated using the Student’s t-test or analysis of variance (ANOVA) as indicated. All statistical tests were performed with SPSS 17.0 (SPSS, Inc., Chicago, IL) with a level of significance of P < 0.05.

## Supplementary Material

Supplementary Figures
