# Supplementary material for: Insulin impedes osteogenesis of BMSCs by inhibiting autophagy and promoting premature senescence via the TGF-β1 pathway
Source: Aging (Albany NY). 2020 Feb 3;12(3):2084–100. doi: 10.18632/aging.102723 (PMC7041775; doi:10.18632/aging.102723)
Supplement: Supplementary Figures [file aging-12-102723-s001..pdf]

SUPPLEMENTARY TABLES

Supplementary Table 1. The demographic data and the specified parameters of donor.

	Gender	Age (years)	Fasting blood glucose (mmol/L)	HbA1c (%)	Fasting insulin (mIU/L)
**Healthy Donor 1**	male	55	5.3	5.0	8.48
**Healthy Donor 2**	male	46	5.2	5.6	9.8
**Healthy Donor 3**	female	50	5.6	5.2	9.3
**T2DM Donor 1**	male	48	6.3	6.7	10.6
**T2DM Donor 2**	female	51	7.3	7.0	9.48
**T2DM Donor 3**	female	47	6.8	6.6	10.8

Supplementary Table 2. Primers Used in Real Time PCR analysis.

Gene	Primer sequence (5′-3′)	Product size (bp)	Tm (˚C)
ALp	F:5-GAGATGGTATGGGCGTCTC-3<br>R:5-GTTGGTGTTGTACGTCTTGGA-3	143	61.6
Runx2	F:5- CAAGTGGCCAGGTTCAACGA-3<br>R:5-GGGACCGTCCACTGTCACTTTAATA-3	141	56.9
Ocn	F: 5-TGCAAAGCCCAGCGACTCT-3<br>R: 5-TTGAGCTCACACACCTCCCTGT-3	159	59.9
Opn	F: 5-GCCGAGGTGATAGCTTGGCTTA-3<br>R: 5- TTGATAGCCTCATCGGACTCCTG-3	136	57.5
β-actin	F:5-GGAGATTACTGCCCTGGCTCCTA-3<br>R:5-GACTCATCGTACTCCTGCTTGCTG-3	150	57.8

Supplementary Table 3. Antibodies used in western blot analysis and immunofluorescence staining.

Western blot:

Antibody/marker	Dilution	Source	Code number
LC3B	1:800	Cell Signaling Technology	#3868
LC3I/II	1:1000	Cell Signaling Technology	#4599
P62	1:1000	Protein Tech	18420-1-AP
TGF-β1	1:1000	Protein Tech	21898-1-AP
P-Smad3	1:1000	Cell Signaling Technology	#8828
Smad3	1:1000	Cell Signaling Technology	#8685
TβR1	1:1000	Abcam	ab31013
TβR2	1:1000	Abcam	ab61213
GAPDH	1:2000	Bioworld	AP0063
β-actin	1:2500	Bioworld	BM0627

Immunofluorescence staining:

Antibody/marker	Dilution	Source	Code number
LC3B	1:200	Cell Signaling Technology	#3868
LC3I/II	1:400	Cell Signaling Technology	#4599
